# Self-sterility, self-incompatibility and xenia: a review of the mechanisms of cross-pollination benefits in animal-pollinated crops

**DOI:** 10.1093/aob/mcaf047

**Published:** 2025-03-20

**Authors:** Stan Chabert, Rachel E Mallinger

**Affiliations:** Department of Entomology and Nematology, University of Florida, Gainesville, Florida, USA; Laboratoire des Interactions Plantes-Microbes-Environnement, Université de Toulouse, INRAE, CNRS, Castanet-Tolosan, France; Department of Entomology and Nematology, University of Florida, Gainesville, Florida, USA

**Keywords:** Animal-pollinated crops, cross-pollination, early-acting inbreeding depression, fertilization, fruit quality, fruit yield, pollination requirements, seed quality, seed yield, self-incompatibility, self-sterility, xenia

## Abstract

**Background:**

While there are multiple mechanisms of self-incompatibility (SI), known to promote outbreeding in angiosperms, these are not well synthesized and described across major global crops. This can lead to misinterpretations of biological processes involved in crop pollination, fertilization and fertility, in particular by confusing them with an additional overlooked phenomenon causing self-sterility (SS), early-acting inbreeding depression (EID). Another overlooked mechanism, called xenia, results in increased quality of seeds and fruits through cross-pollination even in self-compatible and self-fertile crops.

**Scope:**

The aim of this review was to describe and synthesize all the known mechanisms of SI and SS encountered in animal-pollinated (zoophilous) crops, and additional mechanisms by which cross-pollination can be beneficial for crop production. All the known zoophilous crops presenting SS, SI or xenia were quantified and described.

**Key results:**

One hundred and thirty-four zoophilous crops were found to be self-sterile, including 52 displaying complete SS and 82 displaying partial SS. We identified all the known mechanisms of SI and SS in these crops, including gametophytic SI, sporophytic SI, heteromorphic SI, late-acting SI and EID. In addition, 58 zoophilous crops were found to display xenia, including 22 that are self-compatible and completely self-fertile. In total, 156 zoophilous crops were identified as benefitting from cross-pollination for the quantity and quality of seed and fruit production.

**Conclusions:**

While previous reviews focused on quantifying the benefit of animal pollinators for crop production, they did not synthesize the mechanisms underlying pollinator dependence for such crops. Our review provides valuable knowledge about crop pollination requirements in general and more particularly the benefits of cross-pollination across crops ranging in self-fertility. This information could help growers make suitable management decisions regarding their field and orchard planting designs, specifically by mixing mutually suitable cultivars in crops displaying SS or SI, or benefiting from xenia.

## INTRODUCTION

Floral adaptations promoting self-sterility (SS) and outbreeding have been known since Darwin’s works (McClure, 2009; Barrett, 2010). The term ‘self-incompatibility’ (SI) appeared in the early 20th century and progressively became established during the first half of the 20th century as the mechanism of self-rejection involving both the female sporophyte (pistil) and male gametophyte (pollen) (Stout, 1917; de Nettancourt, 2001; McClure, 2009). SI is now an established term that can be often confused with SS; however, the two terms are not interchangeable as a species can be self-compatible while still being self-sterile as there is another, often overlooked mechanism, early-acting inbreeding depression (EID), responsible for SS.

EID is the consequence of inbreeding depression expressed particularly early in the plant life cycle, at the stage of embryo development, and results in partial to full embryo abortion (Krebs and Hancock, 1990; Husband and Schemske, 1995, 1996; Byers and Waller, 1999). However, it is not a consequence of an incompatibility reaction between the female and male gametophytes and thus is not considered a mechanism of SI. EID is responsible for the SS of some crops. For instance, SS of *Vaccinium* species has been attributed to EID since the 1980s (e.g. Hellman and Moore, 1983; Krebs and Hancock, 1988, 1990, 1991; Hokanson and Hancock, 2000) even though it is still often confused with SI (e.g. Danka *et al.*, 2019; Drummond, 2019; Nicholson and Ricketts, 2019; Kendall *et al.*, 2020; Yang *et al.*, 2022; Martin *et al.*, 2024). Though this could be seen as a purely technical discussion about terminology, it has practical consequences for crop pollination management. For instance, if a *Vaccinium* spp. crop is thought to display partial SI (as in Kendall *et al.*, 2020), it may be concluded that the crop only needs more self-pollen deposited on stigmas through more pollinator visits to reach maximum yields. However, because *Vaccinium* spp. displays EID, the goal is to avoid self-pollination as much as possible, and on the contrary to promote cross-pollination as much as possible.

Another phenomenon overlooked in crop pollination literature is xenia (Denney, 1992; Liu, 2008, 2018). This phenomenon leads to an increase in the quality of seeds and fruits through cross-pollination. While completely self-sterile crops are usually managed in fields or orchards interplanted with at least two compatible cultivars to enable cross-pollination, many crops displaying xenia may be self-fertile and self-compatible and thus not planted in diverse cultivar mixes. A recognition of xenia in these crops could lead to altered decisions to mix cultivars in order to increase the quantity and quality of crop production. Thus, it is important to synthesize and document known cases of xenia across global crops.

In addition, understanding the degree of SS in crops, whether partial or full, can inform both future research and planting recommendations. SS broadly can be partial, as is the case for species displaying EID, but it can be also the case for species displaying SI (Levin, 1996; Mena-Ali and Stephenson, 2007; Busch and Schoen, 2008). For crops with partial SS, fertility is said to be limited by pollen quality, as described by Aizen and Harder (2007). As partially self-sterile crops can often produce substantial seeds or fruits following pollination with self-pollen, growers are not necessarily aware that their crop is partially self-sterile and that interplanting their fields or orchards with different cultivars can increase their production through cross-pollination. This is, for instance, the case for northern highbush blueberry (*Vaccinium corymbosum*). While this crop displays EID leading to partial SS, growers in the USA continue to plant their fields with single cultivars (DeVetter *et al.*, 2022). In this review, self-pollen is defined as being pollen deriving from the same flower, from flowers of the same plant, or from plants of the same cultivar.

The aim of this review is to present an overview of all the known mechanisms of SS, including SI and EID, found in the main animal-pollinated crops (hereafter called zoophilous crops), and more generally all the mechanisms by which cross-pollination can be beneficial for the quantity and quality of crop production. This broad objective will accomplish the following sub-objectives: (1) to decrease the confusion that can occur between terms and mechanisms of SS, (2) to increase knowledge of certain overlooked benefits of cross-pollination due to EID or xenia, (3) to improve the management of crop pollination to maximize crop yields and crop quality, and (4) to provide a cross-disciplinary review of the different mechanisms of SS and SI and the benefits of cross-pollination for crop production.

In the following sections, we describe mechanisms for SS, including SI and EID, as well as xenia, and end with a review of the main zoophilous crops reported to display one of those mechanisms. All the main zoophilous crops known to display complete or partial SS, including SI or EID, are listed in Supplementary Data Table S1. This table also includes zoophilous crops that are fully self-fertile if they belong to botanical families or genera with a clear known ancestral SI system. All the main zoophilous crops displaying SI in some cultivars, but including also cultivars that have been made self-compatible through SI breakdown, are listed in Supplementary Data Table S2. Finally, all the main zoophilous crops known to display xenia are listed in Supplementary Data Table S3.

It is important to note that the present review focuses only on the inter-compatibility and inter-fertility of plant genotypes, i.e. on how female sporophyte and gametophyte affect the development and fertilization ability of the male gametophyte (Lora *et al.*, 2016), and on the effect of embryo genotype on its ability to grow until reaching complete maturity. Therefore, this review does not cover the factors that impact the performance of the male gametophyte independently from the female sporophyte and gametophyte, such as how temperature affects pollen germination or pollen tube growth (Coast *et al.*, 2016; Rosbakh *et al.*, 2018; Tushabe and Rosbakh, 2025), or how the level of heterozygosity of the pollen donor affects pollen performance (Stephenson *et al.*, 2001), which actually corresponds to late-acting inbreeding depression and not to EID.

## SELF-STERILITY (SS) AND SELF-INCOMPATIBILITY (SI)

Around 40 % of angiosperm species are estimated to be completely self-sterile (Igić *et al.*, 2008; Raduski *et al.*, 2012; Ferrer *et al.*, 2024). SS is defined as the inability of fertile hermaphrodite seed plants to set viable seeds after self-pollination, i.e. following pollination with self-pollen (de Nettancourt, 2001). SS can be ‘complete’ but also ‘partial’ (Vogler and Kalisz, 2001; Raduski *et al.*, 2012; Ferrer *et al.*, 2024). Reviewing the breeding status of 1238 angiosperm species belonging to 144 botanical families in the literature, Raduski *et al.* (2012) found that 43.9 % of these species have a self-sterility index ≥0.8, the threshold above which species are considered to be completely self-sterile. On the other hand, 25.5 % of these species have an SS index between 0.2 and 0.8, and can thus be considered partially self-sterile, while the remaining 30.6 % displaying an SS index ≤0.2 are considered to be completely self-fertile. Based on the same SS index but analysing a wider pool of 5685 angiosperm species belonging to 222 families, Ferrer *et al.* (2024) found that 37.3 % species are completely self-sterile, 8.1 % are partially self-sterile and the remaining 54.6 % are completely self-fertile. Ferrer *et al.* (2024) also found similar proportions among the cultivated species, with 51.2 % of these species showing complete self-fertility, 11.3 % partial SS and 37.5 % complete SS. Evolutionarily, SS enables plants to avoid the fitness costs of inbreeding and increases their diversification rate, increasing the potential for adaptation to changing environments (Charlesworth and Charlesworth, 1987; Husband and Schemske, 1996; Wright *et al.*, 2013). It is generally observed that woody, perennial and zoophilous plant species are more likely to display SS compared with herbaceous, annual and anemophilous (wind-pollinated) species (Barrett, 1998; Igić *et al.*, 2008; Raduski *et al.*, 2012; Friedman, 2020).

SS encompasses two different processes: SI and EID. There are several types of SI, with different genetic and molecular mechanisms and with an incompatibility reaction occurring at different locations within the pistil. These different types of SI include: the homomorphic gametophytic SI (GSI), the homomorphic sporophytic SI (SSI), the heteromorphic sporophytic SI (HSI), which includes the homomorphic sporophytic diallelic SI (DSI), and the late-acting SI (LSI), the latter also called ovarian SI. SI has been long thought to be exclusively pre-zygotic, i.e. preventing ovule fertilization with self-pollen (Heslop-Harrison, 1975; review in de Nettancourt, 2001; Igić *et al.*, 2008; Ferrer and Good, 2012). But LSI can act pre- or post-zygotically, and independently from EID (Seavey and Bawa, 1986; Sage *et al.*, 1994; Gibbs, 2014; see section on LSI).

SS is polyphyletic, with multiple origins (Igić *et al.*, 2008). It appeared early in the evolutionary history of angiosperms, as it was observed in basal angiosperms with a large diversity of SS systems (Schroeder and Weller, 1997; Endress, 2001; Allen and Hiscock, 2008; Thien *et al.*, 2009). Late-acting forms of SS (LSI and EID) are more widespread among basal angiosperms, and are more basal in this group than other forms of homomorphic SI occurring in the stigma, suggesting that late-acting SS is the ancestral condition of SS (Pontieri and Sage, 1999; Bernhardt *et al.*, 2003; Sage and Sampson, 2003; Koehl *et al.*, 2004; Hristova *et al.*, 2005; Allen and Hiscock, 2008; Gibbs, 2014). In addition, there is real uncertainty that self-compatibility (SC) is the ancestral state of the angiosperm mating system, raising the possibility that SS broadly or SI in particular is the ancestral state (Allen and Hiscock, 2008).

Losses and gains of SS occurred multiple times during the evolutionary history of angiosperms, explaining why we can observe some closely related families possessing completely different and unrelated SS systems, while other more distantly related families share the same type of SS (Weller *et al.*, 1995; Allen and Hiscock, 2008; Igić *et al.*, 2008). In general, SS systems are shared within botanical families as a monophyletic ancestral character, although some lineages can display two or more SS systems within the same family (e.g. Brassicaceae: Busch *et al.*, 2008; Chantha *et al.*, 2013, 2017; Durand *et al.*, 2020; Fabaceae: Supplementary Data Table S1; Uyenoyama, 1995; Allen and Hiscock, 2008; Igić *et al.*, 2008; Gibbs, 2014; Delaney and Igić, 2022; Polemoniaceae: Ferrer and Good-Avila, 2007). Overall, some new forms of SS appeared independently at least 35 times in angiosperm history (Igić *et al.*, 2008; Ferrer and Good, 2012). On the other hand, mutations and duplications of the *S*-locus region, the chromosomal region responsible for some types of SI (see section on GSI and SSI), causing the loss of SI, can occur very easily, explaining a high frequency of transitions to SC that vastly outnumbers SI gains in angiosperm history, especially recently towards the end of lineages (Stone, 2002; Igić *et al.*, 2004, 2008; Robertson *et al.*, 2011).

Despite frequent transitions to SC, the high frequency of SS observed among the current angiosperm species suggests that SS provides a macroevolutionary advantage compared with self-fertility (Igić *et al.*, 2004, 2008; Goldberg *et al.*, 2010; Ferrer and Good, 2012). This macroevolutionary advantage most probably comes from the higher net diversification rates observed in SS lineages compared with the self-fertile lineages (Igić *et al.*, 2008; Goldberg *et al.*, 2010). Although self-fertility can be advantageous in the short term due to reproductive insurance, it may cause an increased risk of extinction owing to a reduced potential for adaptation and appear as an ‘evolutionary dead end’ (Barrett, 2013; Wright *et al.*, 2013). In the short term, self-fertility can also lead to an ‘evolutionary suicide’ as the resulting inbreeding depression may cause extinction (Abu Awad and Billiard, 2017; Cheptou, 2019). For the lineages that had adopted the *S*-RNase GSI system, the reversible transition between SI and SC made possible by the mechanism of non-self-recognition specific to this system may have contributed to their adaptation to diverse and fluctuating environments (Fujii *et al.*, 2016).

## HOMOMORPHIC SELF-INCOMPATIBILITY: GSI AND SSI

Homomorphic SI is controlled by a single *S*-locus region, composed of at least two tightly linked loci encoding both the pollen (male) and pistil (female) *S*-determinants (Allen and Hiscock, 2008; Gibbs, 2014; Goring *et al.*, 2023). The *S*-haplotypes are extremely polymorphic, with often <50 % amino acids shared between alleles, the number of *S*-alleles reaching up to 68 for instance in Asian pear (*Pyrus pyrifolia*; Zhang and Gu, 2019).

**Fig. 1.**
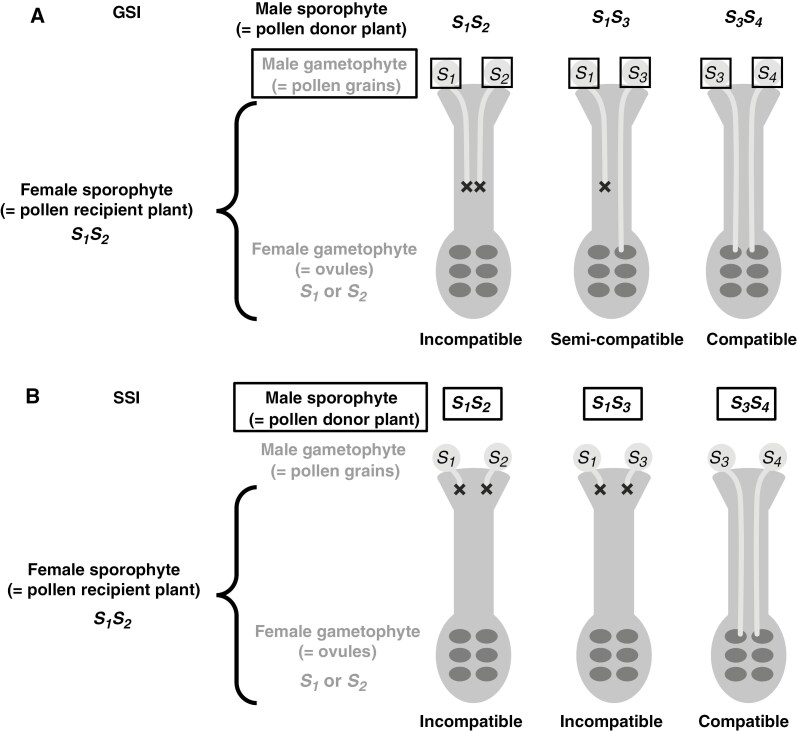
(A) Homomorphic gametophytic self-incompatibility (GSI) and (B) homomorphic sporophytic self-incompatibility (SSI). The boxes around the text show whether the male gametophyte or male sporophyte determines the incompatibility reaction. The crosses show the location of the incompatibility reaction when it occurs. *S*_*1*_, *S*_*2*_, *S*_*3*_ and *S*_*4*_ are examples of alleles of the *S*-locus region, which controls the incompatibility reaction. Figure partly redrawn from McClure and Franklin-Tong (2006). © 2006, Springer-Verlag

Incompatibility caused by homomorphic SI can be gametophytic (= GSI) or sporophytic (= SSI). In GSI, the incompatibility reaction occurs when the *S*-allele carried by the male gametophyte (= pollen) provided by the pollen donor corresponds to at least one of the two *S*-alleles carried by the pollen recipient (McClure and Franklin-Tong, 2006). In this system, a pollen donor sharing the same two *S*-alleles as the pollen recipient will have all its pollen grains rejected by the pistil of the pollen recipient, resulting in complete incompatibility (Fig. 1A), whereas a pollen donor sharing only one *S*-allele with the pollen recipient will have only half of its pollen grains rejected by the pistil of the pollen recipient, resulting in only partial incompatibility of the pollen donor with the pollen recipient (Fig. 1A; e.g. in the apple tree; Schneider *et al.*, 2005).

In SSI, the incompatibility reaction is triggered by the contact of the pollen coat with the stigma (Hiscock and Tabah, 2003). Because the pollen coat contains both of the two *S*-alleles of the pollen donor, derived from the degradation of the anther tapetum late in pollen development, the incompatibility reaction occurs for every pollen grain of the pollen donor as soon as the pollen donor shares at least one *S*-allele with the pollen recipient (Fig. 1B). There is thus no semi-compatibility. This system is complicated by a system of dominance relationships among *S*-haplotypes, and such relationships can occur independently for pollen and stigma. When an *S*-allele is recessive (class II *S*-haplotype), its effect is masked by a dominant *S*-allele (class I *S*-haplotype), and consequently it does not elicit an incompatibility reaction between any two individuals sharing it, thereby resulting in full compatibility. On the other hand, if the *S*-alleles are co-dominant, the mating is incompatible. The relative frequencies of recessive and dominant *S*-alleles within a wild population reflect a dynamic balance between reproductive assurance, favoured by recessive *S*-alleles, and the benefits afforded by outbreeding, namely avoidance of inbreeding depression, favoured by dominant *S*-alleles (Hiscock and Tabah, 2003). The dominance relationships are mediated by sRNAs expressed by dominant alleles that target and transcriptionally silence the incompatibility male determinant of recessive alleles (Tarutani *et al.*, 2010; Durand *et al.*, 2014).

The SSI system is further complicated by an SI mechanism that is simultaneously gametophytically and sporophytically controlled (GSSI) that can occur in some SSI taxa (Lewis *et al.*, 1988; Zuberi and Lewis, 1988; Lewis, 1994; Suassuna *et al.*, 2003). In these taxa, a second gene, called *G*, gametophytically controlling the SI reaction with only two alleles, is superimposed on the *S*-gene, which controls SI sporophytically. The two genes, *G* and *S*, are linked on the same chromosome and each have their own molecular recognition signal. The two genes are complementary in that both *G* and *S* alleles must be simultaneously matched with the same respective alleles in pollen and pistil to cause the incompatibility reaction, explaining why the incompatibility reaction does not occur when the pollen donor and the pollen recipient share one *S*-allele but a different *G*-allele, and vice versa. A distinctive feature of the *G*-gene is that it is expressed only with some particular *S*-allele combinations that are high in the dominance series. The *G*-gene is probably a relic of a former polyallelic primitive GSI system that has been retained and into which the *S*-gene has been incorporated. GSSI has been clearly demonstrated only in two Brassicaceae species (*Brassica rapa* and *Raphanus sativus*; Lewis *et al.*, 1988; Zuberi and Lewis, 1988) and in one Passifloraceae species (*Passiflora edulis*; Suassuna *et al.*, 2003). It may also occur in the Asteraceae, although that has not yet been clearly demonstrated (Lewis *et al.*, 1988; Lewis, 1994).

Most species displaying GSI present wet stigmas and bicellular pollen (de Nettancourt, 1997; Allen and Hiscock, 2008), with the incompatibility reaction occurring mostly within the style at various distances from the stigma (e.g. in *Pyrus* species; Wang *et al.*, 2017). It is expressed after an apparently normal period of growth through a strong expansion of the outer wall of the incompatible pollen tube. This expansion leads to the burst of the tube tip, rich in callose, into the intercellular spaces of the conducting tissues, forming callose depositions (de Nettancourt, 1997). On the other hand, SSI is frequently associated with dry stigmas and tricellular pollen, and the incompatibility reaction occurs at the stigma surface (Allen and Hiscock, 2008). As for GSI, callose depositions are formed in response to the incompatibility reactions, especially when incompatible pollen grains germinate and the resulting pollen tubes grow on the stigma surface (Elleman and Dickinson, 1994).

### Homomorphic gametophytic self-incompatibility (GSI)

GSI is the most widespread known SI system in angiosperms (Allen and Hiscock, 2008). It evolved independently at least four times (Allen and Hiscock, 2008; Igić *et al.*, 2008), with distinct biochemical mechanisms developed during its evolution (de Nettancourt, 1997). It has been recorded in 37 families (Igić *et al.*, 2008; Liang *et al.*, 2020), and rigorously demonstrated as controlled by a one-*S*-locus, multi-allelic mechanism in 19 families (Gibbs, 2014; Liang *et al.*, 2020; Ollitrault *et al.*, 2021).

There are two known types of molecular mechanisms of GSI, the class III T2/*S*-type ribonuclease-based GSI (called hereafter *S*-RNase GSI) and the Ca^2+^-based GSI (Franklin-Tong and Franklin, 2003; McClure and Franklin-Tong, 2006; Ramanauskas and Igić, 2017; Zhang *et al.*, 2024). The Ca^2+^-based GSI has so far only been identified in Papaveraceae, a family that does not include any known self-incompatible zoophilous crops. The *S*-RNase GSI has been fully elucidated in Rosaceae, Solanaceae and Plantaginaceae but is ancestral to almost all core eudicots. In many lineages, it has been lost and then supplanted by SSI or HSI (Igić and Kohn, 2001; Steinbachs and Holsinger, 2002; Allen and Hiscock, 2008; Vieira *et al.*, 2008; Ramanauskas and Igić, 2017, 2021).

**Fig. 2.**
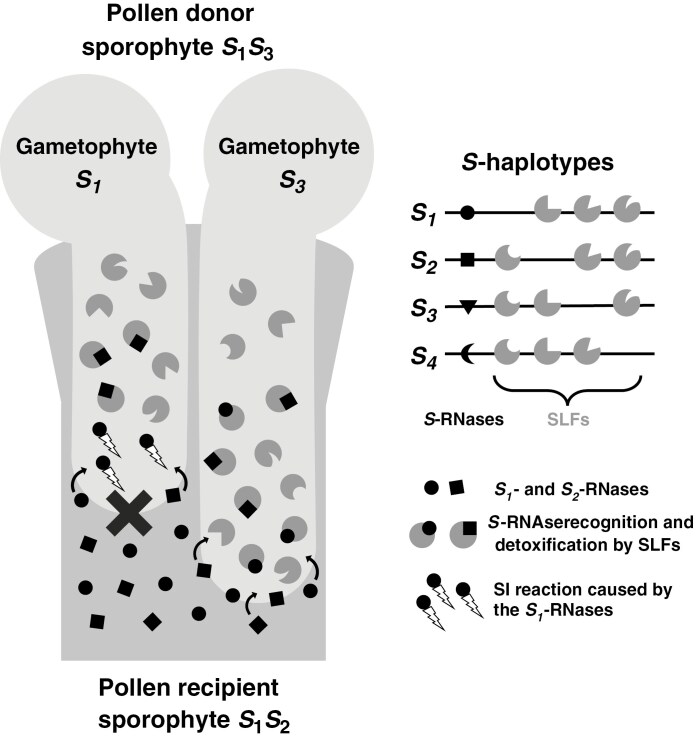
Example of self-incompatibility (SI) reaction with the *S*-RNase homomorphic gametophytic self-incompatibility (*S*-RNase GSI) system. The sporophyte carrying the alleles *S*_*1*_ and *S*_*2*_ receives pollen from the sporophyte carrying the alleles *S*_*1*_ and *S*_*3*_. The *S*_*1*_- and *S*_*2*_-RNases of the pollen recipient are secreted into the pistil extracellular matrix and penetrate the pollen tubes of the gametophytes *S*_*1*_ and *S*_*3*_. Within the pollen tube of the gametophyte *S*_*1*_, the *S*_*2*_-RNases are detoxified by the *S*-locus F-box proteins (SLFs) carried by the haplotype *S*_*1*_ that can recognize them. But the haplotype *S*_*1*_ does not carry the SLFs that can recognize the *S*_*1*_-RNases, allowing the *S*_*1*_-RNases to cause the SI reaction, resulting in the inhibition of the pollen tube, symbolized by a cross. Within the pollen tube of the gametophyte *S*_*3*_, both *S*_*1*_- and *S*_*2*_-RNases are detoxified by the SLFs carried by the haplotype *S*_*3*_ that can recognize them, allowing the pollen tube to grow through the style until the ovary. *S*_*4*_ is another example of a haplotype occurring in the plant population. Figure partly redrawn from Fujii *et al.* (2016), Goring *et al.* (2023) and Zhang *et al.* (2024).

The *S*-RNase GSI acts as a collaborative non-self-recognition system, except in *Prunus* species, for which it acts as a self-recognition system (Fujii *et al.*, 2016). The female *S*-determinants are cytotoxic *s*-RNases that are secreted into the pistil extracellular matrix and penetrate all pollen tubes in a non-specific way during their growth within the style (Fig. 2; de Nettancourt, 1997; Franklin-Tong and Franklin, 2003; Hiscock and McInnis, 2003; Kubo *et al.*, 2010; McClure *et al.*, 2011; Iwano and Takayama, 2012; Fujii *et al.*, 2016; Goring *et al.*, 2023; Zhang *et al.*, 2024). Within the cytoplasm of pollen tubes, the male *S*-determinants, composed of *S*-locus F-box proteins (generally called SLFs), recognize and detoxify all *s*-RNases that are not encoded by the same *S*-allele that encodes for the SLFs via a ubiquitin proteasome pathway. On the other hand, the *s*-RNases that are encoded by the same *S*-allele that encodes for the SLFs are not recognized by the SLFs and can therefore degrade the pollen tube RNA, leading to the bursting of the tube tip, the formation of callose depositions, and ultimately to the inhibition of pollen tube growth (Fig. 2). Thus, the incompatibility reaction acts by default in the pistil, and it is prevented only when the *s*-RNases of the pistil are all encoded by *S*-alleles different from the one encoding for the SLFs of the pollen tube. The recognition of multiple non-self-*s*-RNases is made possible by the presence of multiple SLF genes on the *S*-locus, without the gene that encodes the SLFs recognizing the self-*s*-RNases. It is this operation with multiple SLF genes that gives the ‘collaborative’ dimension to the non-self-recognition system.

*S*-RNase GSI can be lost by *S*-gene mutations, *S*-gene duplication (e.g. by autotetrapolyploidization) or interspecific hybridization (de Nettancourt, 2001; Stone, 2002; Ahmad *et al.*, 2022). The mutation of a single nucleotide is enough to lead to a decrease in the level of *S*-RNase production and activity, and thereby to SI breakdown (Stone, 2002; Watari *et al.*, 2007). Gene duplication can cause SI breakdown in the *S*-RNase GSI system because of the nature of its collaborative non-self-recognition of *S*-alleles (Fujii *et al.*, 2016; Akagi *et al.*, 2022). During male gametogenesis, *S*-heterozygous autotetraploids produce diploid pollen grains with one half *S*-homozygous pollen and the other half *S*-heterozygous pollen. The SLFs of diploid *S*-heterozygous pollen are able to detoxify the *s*-RNases of both *S*-alleles secreted within the pistil, so that the pollen tubes can reach the ovary without triggering an incompatibility reaction. This effect is known as competitive interaction (de Nettancourt, 2001; Fujii *et al.*, 2016). *S*-Heterozygous autotetraploids can be used as pollinizers for all diploid pollen recipients, but not the converse. There are exceptions to this within *Prunus* species, such as sour cherry (*P. cerasus*), that use an *S*-RNase GSI system but with a self-recognition mechanism and for which *S*-heterozygous autotetraploids cannot therefore break down SI (Hauck *et al.*, 2006; McClure, 2009; Tsukamoto *et al.*, 2010; Fujii *et al.*, 2016). Self-compatible cultivars were bred for several zoophilous crops using the three mechanisms of *S*-RNase GSI breakdown: *S*-gene mutation, *S*-gene duplication and interspecific hybridization (Supplementary Data Table S2). In the evolutionary history of the lineages displaying *S*-RNase GSI, self-compatibility is closely associated with polyploidy. Indeed, it was shown that polyploidization caused SI breakdown (Robertson *et al.*, 2011; Zenil-Ferguson *et al.*, 2019). Other abiotic and biotic treatments can be applied in breeding to physiologically and temporarily cause GSI breakdown, for instance in order to maintain inbred lines or to cross two incompatible genotypes (reviews in de Nettancourt, 2001; Ahmad *et al.*, 2022).

### Homomorphic sporophytic self-incompatibility (SSI)

The SSI system is much less widespread than GSI, being restricted to the core eudicots in 11 families (Allen and Hiscock, 2008; Igić *et al.*, 2008; Gibbs, 2014). It arose independently at least ten times in the core eudicots, probably from self-compatible precursors derived from *S*-RNase GSI, with different molecular mechanisms developed each time (Weller *et al.*, 1995; Allen and Hiscock, 2008; Igić *et al.*, 2008). It appears in families at the end of branches in the more advanced lineages, indicating a more recent origin than GSI (Allen and Hiscock, 2008; Igić *et al.*, 2008). SSI can sometimes be superimposed on GSI, leading to a hybrid GSSI system (detailed above).

SSI has been mainly investigated in the Brassicaceae, and in the *Brassica* genus in particular, in which the incompatibility reaction acts as a self-recognition system (Hiscock and McInnis, 2003; Hiscock and Tabah, 2003; Iwano and Takayama, 2012; Doucet *et al.*, 2016; Fujii *et al.*, 2016; Goring *et al.*, 2023; Zhang *et al.*, 2024). By default, when a compatible *Brassica* pollen grain is deposited onto the stigma, the surface pollen coat extrudes on the stigmatic papilla cell to form a ‘pollen foot’ and triggers localized cellular responses in the stigma to transfer water from the stigma to hydrate the pollen grain, cause germination, and result in the emerging pollen tube penetrating the papilla (Doucet *et al.*, 2016; Goring *et al.*, 2023; Zhang *et al.*, 2024). On the other hand, when an incompatible pollen grain lands on the stigma, the male *S*-determinant, an *S*-locus cysteine-rich peptide ligand (SCR) located in the pollen coat, binds to the female *S*-determinant, an *S*-receptor kinase (SRK) located in the plasma membrane of the papilla, if they are encoded by the same *S*-allele. The two *S*-determinant genes are tightly linked on the *S*-locus and highly polymorphic, each *S*-haplotype encoding a matching ligand–receptor pair. The binding of SCRs to SRKs leads to the disruption of papilla secretion and of the cellular functions required for pollen hydration and germination through a complex molecular pathway (Doucet *et al.*, 2016; Goring *et al.*, 2023; Zhang *et al.*, 2024). The molecular mechanisms are less well known in the Asteraceae and Convolvulaceae families, but it appears from preliminary knowledge that these mechanisms are different from those described for *Brassica* (Hiscock and Tabah, 2003).

As for GSI, SSI can be broken down by *S*-gene mutations (de Nettancourt, 2001; Sherman-Broyles and Nasrallah, 2008; Ahmad *et al.*, 2022). But contrary to *S*-RNase GSI, SSI cannot be broken down by polyploidization because of its mechanism of self-recognition of *S*-alleles. Allotetraploidy is nevertheless well associated with self-compatibility in the Brassicaceae, due to polyploidization events that have followed SSI breakdown among diploids in their evolutionary history (Novikova *et al.*, 2023). This is for instance the evolutionary history of oilseed rape (*Brassica napus*), which is a self-compatible allotetraploid resulting from the hybridization of two species displaying SSI, *B. rapa* and *B. oleracea* (Supplementary Data Table S1). Sunflower (*Helianthus annuus*), a species displaying SSI in the wild, has been made largely self-compatible in its domesticated form following crop breeding (Supplementary Data Table S1). And as with the GSI system, other abiotic and biotic treatments can be applied to physiologically and temporarily cause SSI breakdown (de Nettancourt, 2001; Ahmad *et al.*, 2022).

## HETEROMORPHIC SPOROPHYTIC SELF-INCOMPATIBILITY (HSI)

HSI has a scattered distribution among different angiosperm lineages, belonging to at least 34 families. It has evolved at least 152 times independently at the genus level, probably from self-compatible precursors, but this is a minimum estimate because of the likelihood of multiple origins within some genera (Weller *et al.*, 1995; Barrett and Shore, 2008; Igić *et al.*, 2008; Naiki, 2012; Barrett, 2019; Simón-Porcar *et al.*, 2024). HSI appeared later than homomorphic SI and LSI in the evolutionary history of angiosperms (Ferrer and Good, 2012).

**Fig. 3.**
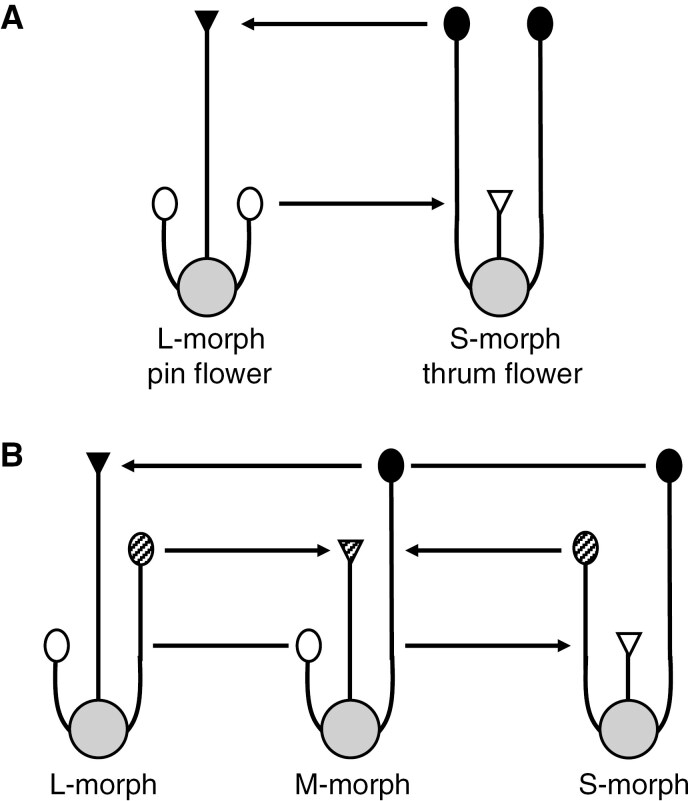
The two forms of heteromorphic sporophytic self-incompatibility (HSI): (A) distyly and (B) tristyly. The arrows show the transport of cross-pollen from anthers towards stigmas of equivalent height favoured by the heterostyly. Figure redrawn from Barrett and Shore (2008). © 2008, Springer-Verlag Berlin Heidelberg

HSI is a sporophytically controlled SI system, coupled with a floral polymorphism called heterostyly or reciprocal herkogamy (Barrett and Shore, 2008; Barrett, 2019). The reciprocal herkogamy prevents pollen from being wasted on incompatible stigmas (Simón-Porcar *et al.*, 2024), while the physiological SI prevents female fitness from being affected by inbreeding depression. Species displaying this system are composed of distylous or tristylous flowers, with most taxa displaying distyly and only four families displaying tristyly (Allen and Hiscock, 2008). Distyly is characterized by flowers with long-style morphs, also called L-morphs or pin flowers, and by flowers with short-style morphs, called S-morphs or thrum flowers (Fig. 3A). These flowers have reciprocal anther positions relative to the stigma: L-morphs bear short stamens, while S-morphs bear long stamens. Tristyly is composed of flowers with long-, mid- or short-style morphs, with two whorls of stamens (Fig. 3B). Mid-style morphs are also called M-morphs. As for distyly, the anthers of each morph are located at heights corresponding to the stigma levels of the two other morphs. Crosses between plants of the same morph, within distylous or tristylous species, are inter-incompatible, so that there is self- and intra-morph incompatibility. Heterostyly is accompanied by ancillary polymorphism of pollen size and stigmatic papilla length: anthers and stigmas located at low positions within flowers have, respectively, pollen grains and papillae with smaller size compared with the anthers and stigmas located at higher positions. This ancillary polymorphism is supposed to increase the probability of stigmas capturing only compatible pollen grains from the other(s) morph(s). Stigmas of the L-morphs usually capture more pollen, but also a higher proportion of intra-morph incompatible pollen, as compared with the S-morphs.

The incompatibility reaction can occur either in the stigma, the style or the ovary, and with little consistency regarding the location of this reaction within families or even between flower morphs within some species (de Nettancourt, 1997). This reaction is often incomplete within each of the three pistil compartments, and needs reactions in all three locations to reach complete incompatibility. Incompatibility reactions in each compartment include partial adhesion and hydration of the incompatible pollen on the stigma, partial ability of incompatible pollen to germinate and penetrate the stigma, and partial ability of the incompatible pollen tubes to reach the ovules. HSI is not based on a dedicated self/non-self-recognition system as with the GSI and SSI systems, but it rather depends on matching physiological adaptations between the pollen tubes and the stylar environment (Kappel *et al.*, 2017; Shore *et al.*, 2019). HSI can lead to complete or partial SS, with different possible levels of partial SS between species, between plants or even between floral morphs within the same species (Barrett and Shore, 2008; Barrett, 2019). Within-morph pollination was, for instance, found to be more self-fertile between M-morphs compared with L- and S-morphs in several unrelated tristylous species.

Distylous HSI is controlled by a hemizygous region of several genes of the *S*-locus leading to a dominant *S*-haplotype encoding for S-morph when present, and to a recessive *s*-haplotype encoding for L-morph when absent (Li *et al.*, 2016; Kappel *et al.*, 2017; Barrett, 2019; Shore *et al.*, 2019; Gutiérrez-Valencia *et al.*, 2022; Fawcett *et al.*, 2023; Yang *et al.*, 2023; Zhao *et al.*, 2023). The *S*-genes and molecular pathways responsible for the SI reaction have started to be elucidated recently in the *Primula* and *Turnera* genera (Barrett, 2019; Shore *et al.*, 2019; Matzke *et al.*, 2021; Henning *et al.*, 2022; Huu *et al.*, 2022; Zhang *et al.*, 2024). In both genera, the presence of analogous *S*-genes in S-morphs inactivates brassinosteroids in the pistil. Brassinosteroids are phytohormones regulating cell expansion, triggering both the elongation of L-morph styles and the growth of S-morph pollen tubes. Thus, the presence of these *S*-genes represses both style elongation and the growth of S-morph pollen tubes in *Primula* (Huu *et al.*, 2022; Zhang *et al.*, 2024). In *Turnera*, the different levels of brassinosteroids in S- and L-morphs were not found to directly control the SI reaction, but are thought to act indirectly through the regulation of gene expression in the pistil, causing different physiologies of the stigma and stylar transmitting tract between the two morphs (Shore *et al.*, 2019; Matzke *et al.*, 2021; Zhang *et al.*, 2024). Another *S*-gene was found to trigger auxin synthesis in stamens in *Turnera*, resulting in different auxin levels between the two morphs, and ultimately determining pollen size and male mating type in each morph (Shore *et al.*, 2019; Henning *et al.*, 2022; Zhang *et al.*, 2024).

### Homomorphic sporophytic diallelic self-incompatibility (DSI)

SI and heterostyly can be genetically decoupled on the hemizygous supergene of the HSI system, so that SI is maintained but heterostyly is lost, as seen in several species of the Oleaceae family, including olive (*Olea europaea*; Castric *et al.*, 2024; Raimondeau *et al.*, 2024). These species thus show homomorphic sporophytic SI like SSI, but with only two mutually incompatible phenotypes. This form of ‘homomorphic HSI’ has been called homomorphic sporophytic diallelic SI, or more briefly ‘DSI’ (Saumitou-Laprade *et al.*, 2010, 2017; Vernet *et al.*, 2016). DSI has been described only in the Oleaceae to date, but since its characterization is recent, it may eventually be discovered in other families.

## LATE-ACTING (OVARIAN) SELF-INCOMPATIBILITY (LSI)

LSI has been recorded in 28 families, belonging to nearly all major angiosperm clades, from basal taxa to the more derived eudicot lineages (Allen and Hiscock, 2008; Gibbs, 2014; Ferrer *et al.*, 2024). Although LSI probably represents the basal state of SS along with EID (see section on SS and SI), it evolved independently on numerous occasions at different times during angiosperm diversification. It shows phyletic clustering, i.e. there is a high probability of finding LSI within the same genera or families, and it has often been correlated with a woody perennial habit (Gibbs and Bianchi, 1999; Allen and Hiscock, 2008; Gibbs, 2014).

**Fig. 4.**
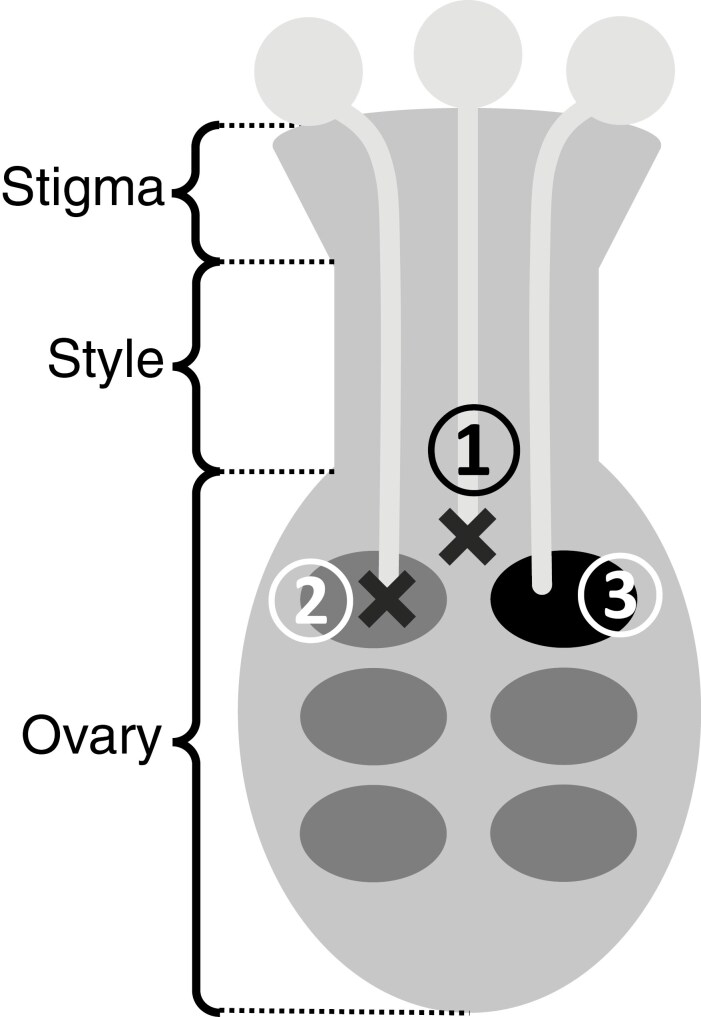
The three locations of the incompatibility reaction caused by late-acting self-incompatibility (LSI): (1) in the ovary, before the pollen tubes penetrate the ovules; (2) in the ovule or the micropyle with syngamy failure; and (3) after double fertilization with early embryo abortion (in black). The crosses show the location of the incompatibility reaction.

The incompatibility reaction of LSI occurs in the ovary following pollination with self-pollen or with incompatible cross-pollen in three different ways (Fig. 4): (1) pollen tubes are blocked in the ovary before penetrating ovules, (2) pollen tubes penetrate ovules, but they are arrested within the micropyle or there is a failure of syngamy following discharge of the male gametes into the embryo sac, or (3) syngamy occurs with double fertilization but embryo development is inhibited (Seavey and Bawa, 1986; Sage *et al.*, 1994; Gibbs, 2014). The first two processes correspond to pre-zygotic LSI, while the third one corresponds to post-zygotic LSI.

For pre-zygotic LSI, it is often observed that self-pollen tubes grow in the ovary or penetrate ovules more slowly compared with cross-pollen tubes (Seavey and Bawa, 1986; Sage *et al.*, 1994; Gibbs, 2014). In some species, ovules may degenerate or lose the ability to attract pollen tubes following the deposition of self-pollen onto the stigma and during the time it takes self-pollen tubes to grow in the style. This early ovule degeneration may be triggered by the spread of hormones through the pistil following self-pollination and the growth of self-pollen tubes in the style.

In post-zygotic LSI species, ovules usually degenerate at the same rate whether fertilized by incompatible pollen or not fertilized at all, and pistils are generally abscised a short time after incompatible pollination, either at the same time as unpollinated pistils or shortly after (Seavey and Bawa, 1986; Sage *et al.*, 1994; Gibbs, 2014). The embryos fertilized by incompatible pollen abort at an early uniform stage of development, typically after the first division of the primary endosperm nucleus, although endosperm divisions can reach up to eight nuclei in some species, or zygotes can completely fail to divide in some other species. The incompatibility reaction is thought to occur spatially between the incompatible pollen tube and the integuments of the ovule. The ovule needs an appropriate stimulation from a compatible pollen tube to be able to develop after fertilization, and this does not occur with an incompatible pollen tube. Recognition and rejection of the incompatible pollen tubes may also be temporally and spatially separated, the recognition occurring for instance at a pre-zygotic stage while the rejection occurs post-zygotically (Sage and Sampson, 2003). For all three processes, the incompatibility reaction may be complete or partial, equivalent to complete or partial SS.

However, the genetic and physiological mechanisms underlying these phenomena are not yet fully understood. LSI is hypothesized to be controlled by multiple loci, contrary to the GSI and SSI systems, which are mostly controlled by a single *S*-locus (Sage *et al.*, 1994; Allen and Hiscock, 2008). But like GSI and SSI, LSI can be gametophytically controlled (G-LSI), sporophytically controlled (S-LSI), or gametophytically–sporophytically controlled (GS-LSI) (examples in Supplementary Data Table S1). It has been proposed that the incompatibility interaction often occurs directly between the gametophytes, i.e. between the pollen tube and the ovule, unlike in the GSI and SSI systems, where it occurs between the male gametophyte (pollen) and the female sporophyte (pistil). Recently, two loci have been identified controlling LSI in cocoa (*Theobroma cacao*; Lanaud *et al.*, 2017), and genetic markers were also identified to help select compatible pollinizers in this crop (Royaert *et al.*, 2011; da Silva *et al.*, 2016).

Consequently, in species presenting post-zygotic LSI or certain forms of pre-zygotic LSI, ovules can be pre-empted following incompatible pollination and can be made unavailable for any concomitant or subsequent pollination by compatible cross-pollen. Ovule pre-emption by incompatible pollen tubes is called ‘ovule usurption’ or ‘ovule discounting’. To maximize fruit and seed production of these species, it is therefore important to avoid incompatible pollination as much as possible to allow for compatible cross-pollination and fertilization. In addition, as cross-pollen donors can still be incompatible, as in the GSI and SSI systems, a careful choice of the pollinizers should be made in crops displaying LSI to be sure that they are compatible with the pollen recipients and to avoid ovule discounting (e.g. cocoa; Vansynghel *et al.*, 2023; kola nut, *Cola nitida*; Nyadanu *et al.*, 2021, 2023*a*, *b*).

## EARLY-ACTING INBREEDING DEPRESSION (EID)

Inbreeding depression is a decrease in fitness-related traits, i.e. the traits related to survival, growth and fertility, of offspring resulting from the mating of two related individuals or following the reproduction of an individual with itself for species that can self-fertilize, such as self-compatible plants (Byers and Waller, 1999; Keller and Waller, 2002; Charlesworth and Willis, 2009; Hedrick and Garcia-Dorado, 2016). It is caused by the expression of a few recessive lethal alleles or by an accumulation of multiple partly recessive weakly deleterious alleles, called genetic load, in the phenotype of inbred individuals. These recessive lethal or deleterious alleles are usually masked by their dominant counterparts in the phenotype of outbred heterozygous individuals, but they become unmasked in the phenotype of individuals made homozygous by inbreeding. Lethal or deleterious alleles are not all spontaneously recessive, but natural selection selects against these alleles when they are dominant. However, they can survive and be transmitted from generation to generation in heterozygous individuals in recessive form. Genetic load can be purged from inbred populations through natural selection, thereby decreasing inbreeding depression, especially for early-expressed traits (Husband and Schemske, 1996; Byers and Waller, 1999; Keller and Waller, 2002; Charlesworth and Willis, 2009; Hedrick and Garcia-Dorado, 2016). Long-lived perennial and outcrossing species usually carry higher genetic loads than short-lived annual and selfing species (Sage *et al.*, 1994; Husband and Schemske, 1995, 1996; Byers and Waller, 1999), and domestication was found to increase the frequency of deleterious mutations of crops compared with their wild relatives, especially for clonally propagated crops (Gaut *et al.*, 2018; Fernie and Yan, 2019). This is because (1) selfing species intrinsically have a higher likelihood of inbred populations that expose their genetic load more frequently to purging through natural selection (Husband and Schemske, 1995, 1996), and (2) individuals of long-lived perennial species accumulate more genetic load with age than individuals of short-lived species (Klekowski and Godfrey, 1989). Selfing species express more inbreeding depression late in their life cycle, at the stage of growth or reproduction, whereas outcrossing species can express it at both stages, either early for seed production, or late (Husband and Schemske, 1996). When alleles that accumulate with inbreeding are lethal, a few homozygous loci are enough to cause the death of individuals. But when alleles are weakly deleterious, inbreeding depression gradually increases as the inbreeding coefficient increases and more loci become homozygous. In other words, when alleles are weakly deleterious fitness gradually decreases with an increase in the inbreeding coefficient (Keller and Waller, 2002; Charlesworth and Willis, 2009; Hedrick and Garcia-Dorado, 2016).

In comparison with late-acting inbreeding depression, EID is inbreeding depression expressed early in the plant life cycle at the stage of embryo development. It results in the abortion of a part of the embryos due to genetic load (e.g. Herlihy and Eckert, 2002). It is mainly due to recessive lethal alleles located on loci coding for embryo development, whereas late-acting inbreeding depression is generally due to weakly deleterious mutations (Husband and Schemske, 1995, 1996; Keller and Waller, 2002). EID appears most often in long-lived perennial and outcrossing species (Wiens *et al.*, 1987; Husband and Schemske, 1995; reviewed in Hokanson and Hancock, 2000). An ‘over-production’ of ovules is expected in self-compatible outcrossing species to compensate for the partial embryo abortion due to genetic load (Seavey and Bawa, 1986; Porcher and Lance, 2005; Harder and Johnson, 2023). In these species, seed and fruit production will therefore be limited by maternal resources if all the flowers are adequately pollinated and fertilized (Wesselingh, 2007; Harder and Aizen, 2010).

The magnitude of EID, i.e. the proportion of aborted seeds, depends on the zygotic inbreeding coefficient *F*_Z_ and on the inbreeding load (Keller and Waller, 2002; Charlesworth and Willis, 2009; Hedrick and Garcia-Dorado, 2016). In the case of self-fertilization, *F*_Z_ will be equivalent to the inbreeding coefficient of the single parent. But in the case of a cross between two parents, *F*_Z_ depends on both the inbreeding coefficients of the two parents and on the relatedness coefficient between the two parents. When two parents are more closely related to one another, *F*z will be higher, and thus EID can be greater. For example, it has been shown that the number of mature seeds decreases and the number of aborted seeds increases as *F*_Z_ increases in *Vaccinium* species (Hellman and Moore, 1983; Krebs and Hancock, 1988, 1990; Harrison *et al.*, 1993), alfalfa (*Medicago sativa*; Fyfe, 1957; Busbice and Wilsie, 1966; Busbice, 1968; Dessureaux and Gallais, 1969) and wild plant species (Crowe, 1971; Seavey and Carter, 1994). Additionally, the number of mature seeds has been shown to decrease, and the number of aborted seeds to increase, with an increase in the relatedness coefficient between two parents in *Vaccinium* species (Hellman and Moore, 1983; Krebs and Hancock, 1990). A careful choice of the pollen donor should thus be made when a pollinizer is interplanted with a pollen recipient, and should depend on the relatedness coefficient between the pollen donor and the pollen recipient.

Polyploid plants should theoretically be impacted by inbreeding depression at a slower rate as compared with diploid plants, as the recessive lethal or deleterious alleles need to be present in a higher number of loci to be expressed in the phenotype. A recent meta-analysis demonstrated this to be true, but it appears that the buffer effect of polyploidy on inbreeding depression decreases with the time elapsed since the polyploidization event. Inbreeding depression is lower in young polyploid lineages compared with their diploid relatives and counterparts, but it is higher in older polyploid lineages (Clo and Kolář, 2022). Perhaps this can explain why inbreeding depression was found to be lower in tetraploid and hexaploid *Vaccinium* species compared with their diploid counterparts or other diploid *Vaccinium* species, but this effect was not consistent for all tetraploid and hexaploid species (El-Agamy *et al.*, 1981; Hellman and Moore, 1983; Vander Kloet and Lyrene, 1987; Hokanson and Hancock, 2000; Schott, 2000). Furthermore, tetraploid plants of alfalfa were not found to display less inbreeding depression as compared with their diploid counterparts (Fyfe, 1957).

As EID can be easily confused with post-zygotic LSI, the following four criteria can be used to disentangle these two mechanisms (Seavey and Bawa, 1986). (1) Embryos abort at an early uniform stage of development in species displaying post-zygotic LSI, while embryos abort heterogeneously throughout the whole period of development in seeds of species displaying EID, resulting in aborted seeds of very different sizes. (2) The number of mature seeds obtained after self-fertilization should be low and homogeneous among plants of a population presenting post-zygotic LSI, whereas it can be quite variable following self-fertilization among plants of a population displaying EID depending on the genetic load of each individual. (3) Only a fraction of the cross-pollen donors of a population are compatible with a pollen recipient presenting post-zygotic LSI, while all cross-pollen donors should yield about the same number of mature seeds if they have the same relatedness coefficient with the pollen recipient in species displaying EID. (4) Embryos resulting from a fertilization with an incompatible pollen donor should be able to be rescued in tissue culture if the expression of incompatibility does not interfere with their autonomous physiology, while embryos aborting because of genetic load should not be able to be rescued in tissue culture. Additionally, we propose another criterion: (5) the number of mature seeds produced per flower should progressively decrease by self-pollinating successive generations of plants displaying EID, while it should not vary for post-zygotic LSI species.

It is important to note that EID and post-zygotic LSI are not the only mechanisms that can lead to seed and fruit abortion. Flowers require the receipt of enough compatible pollen onto the stigma for ovule fertilization, and embryos and developing fruits require enough maternal resources to grow and reach complete maturity (Harder and Routley, 2006; Harder and Aizen, 2010). Seed and fruit abortion can therefore also arise from a deficit of resources available in the maternal plant (Bos *et al.*, 2007; Wesselingh, 2007).

## XENIA

Xenia is the phenomenon of direct effects of pollen from different sources on the traits of seed and fruit quality, including the embryo, endosperm and maternal tissues, that are expressed during the period from fertilization until seed germination (Denney, 1992). These traits include the size, mass, colour, shape, chemical composition, firmness and developmental timing of seeds and fruits. The mechanisms are not yet well understood, but these effects are thought to arise from (1) the double fertilization of the egg and central cells, (2) transposons inherited both maternally and paternally, (3) phytohormones secreted by both the embryo and endosperm that diffuse out into the maternal tissues of the seed and fruit, and (4) the release of mRNAs and small RNAs by the growing pollen tubes that diffuse out into the maternal tissues (Denney, 1992; Liu, 2008, 2018). As an example of the fourth hypothesized mechanism, it has been shown that small-interfering RNAs produced in the pollen vegetative cell can silence transposable element reporters in the sperm cells, showing an example of RNA-mediated communication between non-germ and germ cells (Martínez *et al.*, 2016; Li *et al.*, 2024).

While fruit size, mass and shape are traditionally included in the definition of xenia, it is important to clearly separate traits arising from fertility and xenia in multi-seeded fruits. When the embryos and seeds develop after fertilization, they secrete phytohormones, mostly auxins but perhaps also gibberellins and cytokinin, which diffuse out into the surrounding maternal tissues and promote cell division and extension during the fruit growth stage (Nitsch, 1952, 1953; Crane, 1964; Ozga and Reinecke, 2003; McAtee *et al.*, 2013; Kumar *et al.*, 2014; Li *et al.*, 2022). This explains why a positive correlation has been found in multiple species between the number and distribution of seeds and fruit mass and shape. In these cases, the effect of the pollen source on fruit mass and shape arises directly from a fertility effect and would not be considered an example of xenia. To disentangle whether xenia or fertility is responsible for fruit mass and shape, one can test if fruit mass and shape differ across different pollen sources even if they result in the same number of mature seeds, which would indicate xenia.

The effect of the pollen donor on seed traits and development can be explained by standard Mendelian inheritance or by genomic imprinting (Li and Berger, 2012; Gehring, 2013; Bai and Settles, 2015; Dai *et al.*, 2022; Khouider and Gehring, 2024). Genomic imprinting is the asymmetrical expression of alleles through epigenetic mechanisms in the endosperm and embryo during seed development, depending on whether the alleles are maternally or paternally inherited (Gehring, 2013; Bai and Settles, 2015; García-Aguilar and Gillmor, 2015; Khouider and Gehring, 2024). Imprinted genes thus express either the maternal or paternal allele and are classified as maternally expressed genes (MEGs) or paternally expressed genes (PEGs). Some MEGs were found to control seed size, shape and lipid content (Xiao *et al.*, 2006; Costa *et al.*, 2012; Fatihi *et al.*, 2013; Yuan *et al.*, 2017; Zhou *et al.*, 2021; Ando *et al.*, 2023), and some PEGs were found to control starch content and seed size (Xiao *et al.*, 2006; Yuan *et al.*, 2017; Dai *et al.*, 2022). Currently, the most widely accepted explanation of imprinting evolution is the parental conflict theory, also called kinship theory (Bai and Settles, 2015; Khouider and Gehring, 2024). According to this theory, PEGs are expected to allocate maternal resources to promote seed size at the expense of seed set to maximize paternal fitness, while MEGs are expected to promote seed set at the expense of seed size by equally distributing maternal resources among all offspring to maximize maternal fitness (Haig, 2013).

## REVIEW OF SELF-STERILITY, SELF-INCOMPATIBILITY AND XENIA IN THE MAIN ANIMAL-POLLINATED CROPS

Our review is based on the list of zoophilous crops provided by McGregor (1976), Klein *et al.* (2007) and Siopa *et al.* (2024). These crops are grown for their fruits and/or seeds to be used as food and goods, excluding species used for other purposes such as floriculture unless these plants are propagated through seeds. This list is based on all of the species listed by the Food and Agriculture Organization of the United Nations (FAO, 2021) and known to depend at least partially on animal pollination to maximize the production of fruits and seeds (Siopa *et al.*, 2023, 2024). Although zoophilous crops are almost all visited and pollinated by insects (Klein *et al.*, 2007), a few of them are visited and pollinated by animals other than insects, explaining why we refer to ‘zoophilous’ crops here and not only to entomophilous (insect-pollinated) crops, e.g. white-fleshed pitaya (*Selenicereus undatus*) and durian (*Durio zibethinus*), which are pollinated by bats (Valiente-Banuet *et al.*, 2007; Baqi *et al.*, 2022), and feijoa (*Acca sellowiana*), which is pollinated by birds (Ramírez and Kallarackal, 2017).

To identify the crops displaying some level of SS, including SI, xenia or EID from this zoophilous crop list, we reviewed all the information available about the pollination biology of these crops in McGregor (1976), Denney (1992), de Nettancourt (2001; pp. 20–21), Klein *et al.* (2007), Raduski *et al.* (2012), Gibbs (2014), Liu (2018), Muñoz-Sanz *et al.* (2020), Delaplane (2023) and Ferrer *et al.* (2024). We additionally included reviews about the pollination biology of specific crops when they were available (e.g. Cawoy *et al.*, 2009; Ramírez and Davenport, 2013, 2016; Trueman, 2013; Guerra and Rodrigo, 2015; Ramírez and Kallarackal, 2017; DeVetter *et al.*, 2022; Tel-Zur, 2023; Osterman *et al.*, 2024). All the references quoted in this literature were reviewed as well as all more recent articles that cited this literature to have the most up-to-date knowledge about the pollination biology of these crops. We completed this primary literature search by using Web of Science (Clarivate, www.webofscience.com) and searching the Latin name or common name of the crop by associating the terms: ‘pollination’ OR ‘*compatib*’ OR ‘*steril*’ OR ‘xenia’. We identified the relevant articles based on the title first, and then on the abstract (when available in English). We discarded in particular studies that examined mixtures of pollen from different pollen donors (e.g. Quesada *et al.*, 1991) as studying the effect of the pollen source on fertility and seed and fruit quality requires comparing independent treatments of pollen receipt.

To score each crop as completely or partially self-sterile (Supplementary Data Table S1) or as displaying xenia (Supplementary Data Table S3), we first gathered knowledge about partial or complete SS/SI or xenia from the information directly reported in the literature. Specifically, crops were recorded as partially self-sterile if they were explicitly reported as partially self-sterile or self-incompatible in the literature (e.g. cocoa; Chumacero de Schawe *et al.*, 2013; Vansynghel *et al.*, 2023; silflower, *Silphium integrifolium*; Reinert *et al.*, 2020), or if references stated that there was substantial crop production following self-pollination, or that there was a yield decrease following self-pollination as compared with cross-pollination, or that most of the fruits arose from cross-pollination in comparison with self-pollination (e.g. cashew, *Anacardium occidentale*; Wunnachit *et al.*, 1992; Holanda-Neto *et al.*, 2002). Additionally, we categorized crops as partially self-sterile if a substantial proportion of the genotypes were partially self-sterile even if others were completely self-sterile or self-fertile (e.g. feijoa; Ramírez and Kallarackal, 2017; Sánchez-Mora *et al.*, 2023; mango, *Mangifera indica*; Ramírez and Davenport, 2016). If this information was not explicitly stated in the references, we searched the literature to see if information was available comparing outcomes of hand self-pollination versus hand cross-pollination. If this type of experiment was available for a given crop, the crop was scored as partially self-sterile if at least one article reported a fertility variable (fruit set, seed set, multi-seeded fruit mass) with a significantly lower value for the pollination treatment with self-pollen as compared with cross-pollen (e.g. sesame, *Sesamum indicum*; upland cotton, *Gossypium hirsutum*; Stein *et al.*, 2017). On the other hand, crops were scored as completely self-sterile if they were explicitly reported as ‘completely’ or ‘strictly’ self-sterile or self-incompatible in the literature (e.g. common buckwheat, *Fagopyrum esculentum*; Cawoy *et al.*, 2009), or if references stated that self-pollen tubes do not grow through the pistil (e.g. American elderberry, *Sambucus nigra* subsp. *canadensis*; Warmund *et al.*, 2022), or that SI is ‘strong’ with inhibition of self-fertilization (e.g. chicory, *Cichorium intybus*; Lucchin *et al.*, 2008; Palumbo *et al.*, 2023), or if a clear majority of the genotypes were completely self-sterile (e.g. carambola, *Averrhoa carambola*; Knight, 1982). When this information was not directly available, and when studies were available comparing hand pollination with self-pollen versus cross-pollen, crops were scored as completely self-sterile if fertility was 0 when self-pollinated or if the SS index (see section on SS and SI) was typically ≥0.8 (e.g. Brazil nut, *Bertholletia excelsa*; Cavalcante *et al.*, 2012; muntries, *Kunzea pomifera*; Page *et al.*, 2010). We also scored the crop as displaying xenia if at least one article reported a variable of seed or fruit quality (see the exhaustive list provided in the subsection on xenia) with a significant lower value for the pollination treatment with self-pollen as compared with the pollination treatment with cross-pollen. For the variables of seed quality, we did not include seed germination since it is a trait of seed performance, relevant for hybrid seed production (Frankel and Galun, 1977; Havey, 2004; Colombo and Galmarini, 2017), but not for productions of seeds used as food and goods.

When scoring the crops displaying xenia, we did not include crops for which a difference in multi-seeded fruit mass was found between self-pollination and cross-pollination treatments, as this could be due to an SS effect. We included studies only when they found differences in fruit mass between different cross-pollen donors. Differences in fruit mass between cross-pollen donors could also arise from differences in fertility, but in the absence of clear knowledge we decided to classify them as a xenia effect. Other multi-seeded fruit traits, such as developmental timing or firmness, the latter depending on the level of pectin (Van Buren, 1991; Wang *et al.*, 2018), could also be positively correlated with the number of seeds and thus arise from a fertility effect. But to our knowledge this has not yet been shown, and we classified these characteristics as xenia effects as well.

### Self-sterility

Overall, we identified 134 zoophilous crops presenting SS (Supplementary Data Table S1). Among these crops, 52 are completely self-sterile and 82 are partly self-sterile. The Rosaceae, Passifloraceae and Fabaceae families represent most (56 %) of the completely self-sterile crops, while the Rutaceae, Fabaceae and Ericaceae represent 41 % of the partly self-sterile crops. Completely self-sterile crops are mostly composed of tree crops (44 %), followed by herbs (25 %), vines (19 %) and shrubs (12 %). Partially self-sterile crops are also mostly composed of tree crops (39 %), followed by shrubs (32 %), herbs (18 %) and vines (11 %). While interplanting different cultivars is necessary to get significant fruit and seed production for the completely self-sterile crops, it is not necessary for the partly self-sterile crops. However, while planting partly self-sterile crops with a single cultivar can lead to substantial harvests of fruits and seeds, interplanting different cultivars within the same field or orchard can increase and maximize fertility through cross-pollination, i.e. fruit set, seed set and fruit mass, the latter often positively correlated with seed set in multi-seeded fruits due to increased phytohormone production (Nitsch, 1952, 1953; Crane, 1964; Ozga and Reinecke, 2003; McAtee *et al.*, 2013; Kumar *et al.*, 2014; Li *et al.*, 2022).

### GSI

Forty-six zoophilous crops displaying GSI were identified, of which 40 display *S*-RNase GSI. Most of them (34) belong to the Rosaceae or Rutaceae families, while the others belong to the Cactaceae, Fabaceae, Rubiaceae and Solanaceae families (Supplementary Data Table S1; Gibbs, 2014; Liang *et al.*, 2020; Ramanauskas and Igić, 2021). Nearly half of these crops are partly self-sterile, of which a majority (13) are *Citrus* species and show partial self-sterility because of partial parthenocarpy. The eight non-*Citrus* crops displaying GSI with partial self-sterility illustrate that GSI does not systematically inhibit the growth of all incompatible pollen tubes. In these species, a substantial number of incompatible pollen tubes can nevertheless reach the ovary and fertilize an ovule, resulting in partial crop fertility, despite the incompatibility barriers. However, the 22 other crops displaying GSI are completely self-sterile.

### SSI

Nine zoophilous crop species were identified displaying SSI: three Asteraceae, four Brassicaceae, one Convolvulaceae and one Passifloraceae (Supplementary Data Table S1). Among these nine crops, three are also controlled gametophytically (= GSSI), as described in section on GSI and SSI. All these crops are completely self-sterile except two: turnip (*Brassica rapa*), which displays GSSI, and silflower (*Silphium integrifolium*). This means that, as with the GSI system, SSI does not systematically inhibit the germination and growth of all incompatible pollen grains and tubes in all species. The 11 species of the *Passiflora* genus, other than passion fruit (*P. edulis*), known to present partial or complete self-sterility, most probably also display GSSI or SSI, like passion fruit.

### HSI

Only two main zoophilous crops are known to display HSI, both distylous (Supplementary Data Table S1): carambola (Oxalidaceae) and common buckwheat (Polygonaceae). Both species are completely self-sterile.

### LSI

Twenty-one zoophilous crops were identified displaying LSI among the Anacardiaceae, Fabaceae, Fagaceae, Lardizabalaceae, Malvaceae, Myrtaceae, Sapindaceae and Theaceae families (Supplementary Data Table S1). All of them are shrubs or trees, except three Fabaceae herbaceous species. Eleven crops were shown to display pre-zygotic LSI and five were shown to display post-zygotic LSI. Four crops are known to be S-LSI, two Anacardiaceae and two Malvaceae crops, the two Anacardiaceae crops being post-zygotic, and one of the Malvaceae (cocoa) being pre-zygotic GS-LSI. Four crops were found to be pre-zygotic G-LSI, three *Camellia* species (Theaceae) and sunn hemp (*Crotalaria juncea*; Fabaceae). Almost three quarters (15 crops) of the crops displaying LSI are partially self-sterile, the others (6 crops) being completely self-sterile. Pre- and post-zygotic barriers do not correlate to the level of crop SS as both barriers are present in completely or partially self-sterile crops: 2 pre-zygotic LSI crops out of 11 (18 %) were found to be completely self-sterile, as compared with one 1 of 5 (20 %) for post-zygotic LSI crops.

### EID

Eighteen zoophilous crops were identified displaying EID, most of them (15) belonging to the Ericaceae (*Vaccinium* spp.) and Fagaceae (*Castanea* spp.), while three others belong to the Euphorbiaceae, Fabaceae and Sapindaceae (Supplementary Data Table S1). Most of these crops are self-compatible, but the *Castanea* species and alfalfa present EID along with LSI (e.g. Xiong *et al.*, 2019 for Chinese chestnut, *Castanea mollissima*). All the crops displaying EID are partially self-sterile, and all are perennial shrubs or trees except one perennial herbaceous species, alfalfa. Additionally, all present flower traits promoting outcrossing: *Vaccinium* species bear urn-shaped flowers with the corolla aperture turned downwards and with poricidal anthers that require sonication by insects to release pollen, limiting the deposition of self-pollen onto the stigma in these species (DeVetter *et al.*, 2022), *Castanea* species and lychee (*Litchi chinensis*) are duodichogamous (Menzel, 1984; Pauly *et al.*, 2023), and alfalfa is predominantly outcrossed (Parajuli *et al.*, 2021).

### Xenia

Fifty-eight zoophilous crops were recorded displaying xenia, including 22 crops for which no SI, or complete or partial SS, has been demonstrated, i.e. crops effectively or supposedly self-compatible and completely self-fertile (Supplementary Data Table S3). Most of these crops (52 %) are trees, followed by shrubs and vines (17 % each), and herbs (14 %). The pollen donor has been recorded to have effects on fruit set, individual fruit mass, fruit shape, time to ripen, fruit firmness, fruit chemical content and composition (°Brix, acidity, Brix:acid ratio, vitamin C, polyphenols, anthocyanins, volatile compounds, flavours, linalool oxide), fruit colour (epicarp, sarcotesta), seed set, individual seed mass, seed chemical content and composition (sugars, proteins, starch, amylase, vitamin C, oleic acids, fatty acids, glucosinolates, sinapic esters, tocopherols, amygdalin, morphine, narcotine), seed hardness and toughness, seed colour (endosperm, coat) and length of seed appendages. Peony (*Paeonia ostii*) and pomegranate (*Punica granatum*) are also two examples for which it was demonstrated that the pollen donor has an effect on seed set while no mechanism of SI or EID was described (Supplementary Data Tables S1 and S3; Gharaghani *et al.*, 2017; Xie *et al.*, 2017). Perhaps for these species, differences in seed set may be explained by imprinted genes.

## CONCLUSIONS

In total, 156 zoophilous crops were identified as benefitting from cross-pollination for fertility or seed and fruit quality. This includes far more than the number of crops that are completely self-sterile (52), and thus strictly require cross-pollination. Such strictly self-sterile crops are likely already managed for cross-pollination and interplanted with pollinizers in fields/orchards. Some crops displaying partial SS are already planted in mixed cultivar blocks (i.e. interplanted with pollinizers), such as cocoa (Akoa *et al.*, 2021; Vansynghel *et al.*, 2023), mango (Dag *et al.*, 1998; Lucas-García *et al.*, 2021) and kola nut (Nyadanu *et al.*, 2021, 2023*a*, *b*), but this is not the case for all partially self-sterile crops. For instance, macadamia (*Macadamia integrifolia*, *M. tetraphylla* and hybrids between the two species) orchards and highbush blueberry fields (*Vaccinium* spp.) continue to be planted in single-cultivar blocks (Kämper *et al.*, 2021; DeVetter *et al.*, 2022; Trueman *et al.*, 2022), while it has been known for decades that these crops require cross-pollination to maximize yields (macadamia: e.g. Ito and Hamilton, 1980; Sedgley, 1983; reviewed in Trueman, 2013; highbush blueberry: e.g. White and Clark, 1939; Morrow, 1943; Meader and Darrow, 1947; reviewed in DeVetter *et al.*, 2022). Many zoophilous global crops would therefore benefit from being interplanted in mixed cultivar blocks to maximize yield quantity and quality. Cultivar mixture is also an agronomic practice that may help enhance crop productivity and stability against extreme weather events, as well as enhance pest and pathogen control, decrease pesticide inputs, and thereby save on input costs for growers (Jacquet *et al.*, 2022; Chabert *et al.*, 2024).

Whereas the GSI and SSI systems are quite well known and identified in the crop pollination literature, this is less the case for LSI, EID and xenia, which are largely overlooked and can be confused for one another. Furthermore, crops thought or proven to be self-compatible could still see increases in fertility or the quality of seeds and fruits with cross-pollination if there is EID or xenia in those crops. In many, but not all, cases, information on related crops within the same family could shed insight into the level of self-fertility and mechanisms of SS in understudied crops. For instance, 44 crops were found to display partial SS but without the SS mechanism having been identified (all the boxes that were left empty in the Mechanism column in Supplementary Data Table S1).

For each SS system, a careful choice of the pollinizers should be made. For the SI systems, whether GSI, SSI, HSI or LSI, the pollinizers should be compatible with the pollen recipient cultivar. For EID, the pollinizers should theoretically share a low relatedness coefficient with the pollen recipient. For xenia, the genetic and physiological mechanisms are mostly not yet understood, and thus there is no general recommendation for the choice of pollinizer except that it be non-self. Several pollen donors should be tested with each pollen recipient individually, and the pollen donors yielding the best results for a given pollen recipient should be considered for the pollen recipient. The spatial arrangement of cultivars should also be carefully optimized by considering the foraging behaviour of pollinators and the distance to which they are able to disseminate cross-pollen (reviewed in Chabert *et al.*, 2024; see also MacInnis and Forrest, 2020; Anders *et al.*, 2023; Tscharntke *et al.*, 2024).

Finally, there is a crucial difference between GSI, SSI, HSI and pre-zygotic LSI without ovule discounting on the one hand, and pre-zygotic LSI with ovule discounting, post-zygotic LSI, and EID on the other hand. For the first SS systems, it does not matter if self-pollen is deposited onto the stigma, as long as the stigma is not clogged with self-pollen, which can prevent cross-pollen deposition (Wilcock and Neiland, 2002). Successful pollination and fertilization can still occur with cross-pollination following self-pollen deposition provided the stigma is unclogged. On the other hand, for the second set of SS systems, ovules can be pre-empted by self-pollen tubes that reach the ovary before cross-pollen tubes, preventing any further fertilization by cross-pollen, even if cross-pollen is deposited onto the stigma and cross-pollen tubes reach the ovary. For these systems, it is important to avoid pollination by self-pollen as much as possible for the benefit of cross-pollination.

## SUPPLEMENTARY DATA

Supplementary data are available at *Annals of Botany* online and consist of the following. Table S1: list of zoophilous crops displaying total or partial self-sterility, including crops with total self-fertility but belonging to botanical families or genera with a clear known ancestral self-incompatibility system. Table S2: list of zoophilous self-incompatible crops for which self-compatible cultivars were bred through breakdown of homomorphic self-incompatibility. Table S3: list of zoophilous crops for which a xenia effect has been highlighted.

mcaf047_suppl_Supplementary_Tables

## Data Availability

No data were used for the research described in the present article.
